# Foods Contributing to Macronutrient Intake of Women Living in Puerto Rico Reflect Both Traditional Puerto Rican and Western-Type Diets

**DOI:** 10.3390/nu10091242

**Published:** 2018-09-06

**Authors:** Emily Truesdell, Michelle Schelske-Santos, Cruz María Nazario, Rosa V. Rosario-Rosado, Susan E. McCann, Amy E. Millen, Farah A. Ramírez-Marrero, Jo L. Freudenheim

**Affiliations:** 1 Department of Epidemiology and Environmental Health, University at Buffalo, Buffalo, NY 14214, USA; ejt5@buffalo.edu (E.T.); aemillen@buffalo.edu (A.E.M.); 2 Nutrition and Dietetics Program, University of Puerto Rico Rio Piedras Campus, Rio Piedras, San Juan, PR 00925, USA; michelle.schelske@upr.edu; 3 Department of Biostatistics and Epidemiology, Graduate School of Public Health, Medical Sciences Campus, University of Puerto Rico, San Juan, PR 00935, USA; cruz.nazario@upr.edu (C.M.N.); rosa.rosario1@upr.edu (R.V.R.-R.); 4 Department of Cancer Prevention and Control, Roswell Park Comprehensive Cancer Center, Buffalo, NY 14263, USA; Susan.McCann@RoswellPark.org; 5 Department of Physical Education and Recreation, University of Puerto Rico Rio Piedras Campus, Rio Piedras, San Juan, PR 00931, USA; farah.ramirez1@upr.edu

**Keywords:** Puerto Rican diet, Hispanic diet, macronutrient sources, dietary variation sources

## Abstract

Lack of variability in dietary intake within a population makes identification of relationships between diet and disease difficult. Studies in populations with greater interindividual variation can provide important insights. The Puerto Rican diet is in transition from a traditional to a more Western-type diet, resulting in greater interindividual variability. We identified foods contributing to absolute intake and variability in the intake of macronutrients among Puerto Rican women. One hundred women, aged 30–79, residents of San Juan, Puerto Rico, completed three, interviewer-administered, 24-h dietary recalls from which foods contributing to absolute intake and intake variability in intake of energy, fat, protein, carbohydrate and dietary fiber were determined. The overall prevalence of intake of foods was also calculated. Traditional Puerto Rican foods such as legumes, rice, and plantains were important contributors to the intake of calories and macronutrients as were foods more typical of Western diets including white bread and sweetened carbonated beverages. Identification of food sources of nutrients for this population with a diet in transition can contribute to the development of instruments to measure dietary intake and to understand the contribution of diet to the etiology of chronic disease among Puerto Rican women.

## 1. Introduction

In the examination of diet in relation to chronic disease, lack of variability in intake within a population can be a possible explanation for null results. With lower variability, and consequently fewer individuals at the extremes of intake of a particular nutrient or food component, it can be difficult to identify diet-disease relations with that nutrient or food component. If the intake of most individuals in a population is at or above the threshold for the effect of diet on disease risk, then it will appear that there is no association between intake and risk. Examination of populations with greater variation in dietary intake has potential for providing insight into understanding diet and chronic disease relationships, such as for cancer and cardiovascular diseases.

Cancer and cardiovascular diseases are the leading causes of mortality in Puerto Rico, as they are in the United States (USA). Rates in Puerto Rico are lower than in the USA but increasing more rapidly [1,2,3,4,5,6]. The reasons for these differences are not known; diet may be a contributing factor. The diet in Puerto Rico is in transition from a traditional diet to a more Western-type diet [7], resulting in greater inter-individual variability in intake. Identification of foods that are the major contributors to intake of macronutrients in Puerto Rico and foods contributing to variability in nutrient intakes between individuals in the population can provide insight into the diet in this particular population, contribute to the development of culturally-specific food frequency questionnaires and potentially inform the understanding of the contribution of diet to chronic disease etiology among Puerto Ricans.

Studies of foods that contribute to intake and variability in intake within populations have been conducted globally, including in the USA [8,9,10,11,12,13,14,15,16,17,18,19,20,21,22,23,24,25,26,27,28,29]. These studies provide information regarding food practices and have been instrumental in the development of food frequency questionnaires. There are few published studies of this kind from Central and South America or the Caribbean [7,21,22,23,24]. To our knowledge, there are no quantitative analyses of the contribution of various foods to intakes of adults living in Puerto Rico. While there have been studies of foods contributing to nutrient intake, specifically of Hispanic adults residing in the USA [16,25,26,27,28,29], there are differences in geography, culture, and availability of foods in Puerto Rico, with resulting differences in intake, food preferences and consumption habits between Puerto Rican islanders and those living in the continental USA [30].

For this study, we characterized foods contributing to absolute intake and to variability in intake of total energy, protein, carbohydrate, fat, and dietary fiber in the diets of women in Puerto Rico as well as determining the prevalence of consumption of foods in each food category.

## 2. Methods

### 2.1. Study Population

Participants consisted of 100 women who were residents of the San Juan metropolitan area. All women were between the ages of 30 and 79. The participants were selected in two ways. Seventy-five were selected randomly from the population. A list provided by the Estudio Continuo de Salud, a multi-phased probabilistic sample of the general population based on the selection of housing units from census blocks within conglomerates of municipalities in Puerto Rico [31], was used to randomly identify households. Community health workers went to those households and determined if there was a woman who met the eligibility criteria in that household. If there was an eligible woman, she was invited to participate in the study. The remaining twenty-five study participants were recruited from community-based faith organizations in San Juan. Study participants provided informed consent before they participated in the study. The study was conducted in accordance with the Declaration of Helsinki and the protocol was approved by the Institutional Review Boards of both the University of Puerto Rico Medical Sciences Campus and the University at Buffalo.

### 2.2. Data Collection

Three 24-h dietary recalls were collected for each participant. Interviewer-administered recalls were conducted by phone by a trained interviewer. There were 48 interviews on weekend days and 252 on weekdays. Interview calls were unannounced so that the participant would not modify her diet on days when intake was assessed. For each 24-h recall, the participant was asked to recall her dietary intake for the previous day. A double-pass method was used; the participant was first asked to broadly list the foods that she had consumed throughout the day and then subsequently asked to provide more detail about each food item. The participants had been previously provided with a pamphlet with pictures of different serving sizes to aid them in portion estimation. The foods reported by participants were matched to the 160,000 food items in the University of Minnesota Nutrition Data System for Research (NDSR), a database that includes more than 300 Hispanic foods (http://www.ncc.umn.edu/products/). The NDSR includes prompts for information about each food, such as method of preparation, ingredients and serving size. The data base was used to calculate the nutrient content of each food [32].

### 2.3. Grouping of Foods

In the 24-h recalls collected for this study, participants reported 904 different food items. Each food item was assigned an individual food code in the NDSR. Foods were then grouped according to nutrient similarity and manner of consumption (eaten at similar times of day, and prepared with similar methods). Foods that were reported at least 10 times by any study participant were examined separately. We made several exceptions for foods that were unique from other foods, either nutritionally or in terms of ingredients, and which contributed reportable percentages of energy or macronutrient intake when considered alone. A fruit or vegetable was considered separately when five or more total servings were reported; there were few fruits and vegetables that were reported at least 10 times. When fewer than five servings were reported, that item was placed in a group of “other fruits” or “other vegetables”. Mixed dishes were divided into groups based on the type of meat or seafood that they contained. Typically, these dishes were combinations of meat or seafood, a starch such as rice or tuber roots, vegetables, and seasonings. Mixed dishes that consisted of rice and vegetable with no meat or seafood were placed in a separate category. All foods reported were included either individually or in a broader category.

### 2.4. Ranking Foods Based on Intake Contribution

After foods were grouped, we calculated the percent contribution of each group to the intake of each macronutrient.

For each food group, and for each nutrient:

(1) $$\begin{array}{c} \%\;\mathrm{contribution}\;\mathrm{to}\;\mathrm{intake}\;\mathrm{of}\;\mathrm{nutrient}\;\mathrm{by}\;\mathrm{food}\;\mathrm{group}\;i\;\;\\ =\frac{\sum_{j=1}^{100}\sum_{f=1}^{x}\sum_{k=0}^{S_{\mathrm{fj}}}{\mathrm{Nutrient}}_{\mathrm{fjk}}}{\sum_{i=1}^{98}\sum_{j=1}^{100}\sum_{f=1}^{x}\sum_{k=0}^{\mathrm{Sfj}}{\mathrm{Nutrient}}_{\mathrm{fijk}}}\times 100 \end{array}$$

where:

f = food in food group i
= total number of foods in food group i
= Food group, 1, 2…98
= Participants, 1, 2…100
= Reported servings of food f to participant i, 0, 1, 2…
S_fj_ = Servings of fth food consumed by jth person
Nutrient_fijk_ = Amount of nutrient contained in serving k of food f in food group i to participant j.

Once we had calculated the percent contribution of each food group to the intake of each macronutrient, we ranked the foods based on these values and calculated a running total of the contribution of the foods to nutrient intake.

### 2.5. Ranking of Foods Based on Contribution to Variability

In addition to examining the contribution of foods to absolute intake, we also examined food group contributions to variability in intake of each macronutrient, that is, those foods which were most strongly associated with differences in intake. We constructed multiple linear regression models in which the dependent variable was the total intake of the nutrient of interest during the 3 days for which dietary recall data were collected for each participant. Independent variables were the amount of nutrient contributed by each food group during the three days of recall. Food groups were added to the equation in decreasing order of contribution to total intake of the nutrient.

The regression equation is as follows:

(2) $$Y=\alpha +\beta_{1}X_{1i}+\beta_{2}X_{2i}\ldots$$

where:

Y = Total intake of nutrient y for three days of recall for each participant
X_1_ = Total grams of nutrient y from food group 1 consumed during 3 days of recall
β_1_ = Predicted increase in average daily intake of nutrient y associated with a 1 g increase in consumption of nutrient y from food group 1
X_2_ = Total grams of nutrient y from food group 2 consumed during 3 days of recall
β_2_ = Predicted increase in average daily intake of nutrient y associated with a 1 g increase in consumption of nutrient y from food group 2.

For each food group that contributed to the total intake of a nutrient, the correlation of intake of that food group and total intake of the nutrient was calculated, a measure of the strength of the relationship between the intake from a food group and the total intake of the nutrient without adjustment for the other food groups. Cumulative *R*^2^, the portion of between-participant variability in nutrient intake explained by the increasingly large list of food groups in the model, was calculated and reported after each food group was added to the final regression equation.

### 2.6. Calculating Prevalence of Consumption

To gain a better understanding of the commonality of foods in the Puerto Rican diet, we also ranked food groups based on prevalence of consumption. Prevalence of consumption was calculated as follows:

P(x) = (W/N) × 100%(3)

where:

P = Percent of participants who reported consuming ≥1 serving of ≥1 food in food category x
= Number of participants who reported consuming ≥1 servings of foods in food category x
= Total number of participants (100).

Intake contribution, regression and prevalence of consumption analyses were conducted using SAS^®^ version 9.2 (SAS Institute Inc., Cary, NC, USA).

## 3. Results

The mean age of study participants was 56.4, standard deviation 11.9. Mean BMI was 29.2 (SD 5.6). Twenty-four percent of the participants had normal BMI (BMI < 25), 37% were overweight (BMI between 25 and 30) and 39% were obese (BMI > 30.0). Most of the study participants had at least a high school education (4% had less than 12 years of education); the mean number of years of education was 12.4 (SD 3.0). There were 47 participants who reported on their diet on at least one weekend day; there were no differences in intakes of any of the macronutrients for participants reporting on weekend days compared to those reporting only on weekdays (data not shown).

Foods which accounted for 90% of total intake of energy in kilocalories, and grams of fat, protein, carbohydrate, and dietary fiber in the Puerto Rican diet are shown in Table 1, Table 2, Table 3, Table 4 and Table 5. Several foods were particularly important in terms of contribution to absolute macronutrient intake. White breads, rolls and crackers were the largest contributor to both energy intake and carbohydrate intake, and rice was the second largest contributor to intake of both. Cheese was the largest contributor to intake of fat and among the highest contributors to protein intake. Beefsteaks, roasts and burgers were among the major contributors to intake of fat and protein as were chicken and turkey. Plantain dishes were an important contributor to intake of energy, dietary fiber, and carbohydrates. Bean dishes were the largest source of dietary fiber, though they were only the 13th largest contributor to caloric intake and the 11th largest contributor to protein intake.

Also reported in Table 1, Table 2, Table 3, Table 4 and Table 5 are the correlation coefficients, *r*, representing the strength and direction of the relationships between participants’ intakes of foods and their total intakes of the nutrient. Most food groups were positively correlated with nutrient intake, meaning that a greater consumption of that food group was related to a greater intake of the nutrient. Most correlations were weakly positive to moderately positive, between 0.10 and 0.30. Correlations greater than 0.19 were significant at *p* < 0.05. Some food groups were negatively correlated with the intake of a nutrient, although these negative correlation coefficients were small in magnitude. Cumulative *R*^2^, the percent of variability in intake of the nutrient explained by food groups of that rank and higher, is also reported in each table. For most nutrients, foods that were important contributors to intake also explained large percentages of variability in the intake of that nutrient. In some cases, foods that were not important contributors to total intake were large contributors to variability. Generally, these were foods that were good sources of a nutrient but were not commonly consumed by participants. For example, potato chips and other packaged, salted snacks contributed less than 0.5% of total fat intake, but explained an additional 3% of the variability of total fat intake. Pasta dishes accounted for only 2% of energy intake, but explained an additional 6% of the variability in energy intake when added to the regression model.

The prevalence of consumption of each of the food groups is shown in Table 6. Foods which were important contributors to the intake of macronutrients were also generally consumed by most participants. White breads, rolls and crackers were consumed by 82% of participants, cheese by 82%, rice by 72%, plantain dishes by 45% and bean dishes by 68%.

## 4. Discussion

The foods contributing to macronutrients in the reports from this sample of women in the San Juan metropolitan area included important contributions from traditional Puerto Rican foods (e.g., rice, plantains, fish, tuber roots and bean dishes) as well as from foods more characteristic of the USA/Western-type diet (e.g., white bread, rolls and crackers, meats, cheese, cakes, cookies and donuts). Bean dishes were consumed at least once in the 3 days of dietary recall by more than half of the study population and were the largest source of fiber intake. Legumes are also consumed in the USA but are not as important sources of these nutrients [17]. Fish and fried fish, included in the traditional Puerto Rican diet, were consumed at least once by about one third of the women studied, contributing to more than 3% of protein intake but not contributing substantially to energy intake. In the 2007–2010 USA National Health and Nutrition Examination Survey (NHANES), fish consumption was similar [20]. In Puerto Rico, rice was an important contributor to energy and carbohydrate intake. It was commonly seasoned and sometimes combined with vegetables such as onions, peas (mostly the legume, pigeon peas), or tomatoes cooked in canola or corn oil. We divided these dishes into two groups: plain rice, and rice and vegetable dishes. If these two rice groups were combined, they would have been the largest contributor to both energy and carbohydrate intakes. Additionally, many of the mixed dishes also contained rice and vegetables. In the 2003–2006 NHANES, rice was less important, contributing just about 1% of energy intake and 3% of carbohydrate consumption [17]. Plantain, a traditional food in the Puerto Rican diet, was reported as consumed by 45% of participants. It was among the largest contributors to energy, carbohydrates and dietary fiber. Another traditional food, starchy tuber roots were an important source of fiber and a less important source of carbohydrates and energy. About a quarter of participants reported eating at least one serving of tannier or other tuber roots. The largest sources of dietary fiber were traditional Puerto Rican foods. Bean dishes, plantain dishes, and tannier and other tuber roots together contributed more than a quarter of dietary fiber intake and explained about a third of the variability in intake. Foods typical of a Western-type diet such as white bread, rolls and crackers, pasta and French fries contributed smaller amounts of fiber in the diet. In the USA diet, by contrast, about 11% of fiber comes from yeast bread and rolls, while about 8% comes from legumes [17]. The category of white breads, rolls and crackers was the largest contributor to energy intake both in Puerto Rico and among USA adults in the NHANES 2003–2006 data [17]. Soft drinks, an important source of energy intake in the USA (20% of energy for adults 19–50), were not as important in our study population (4%). Beef was a larger contributor to protein (14.5%, adults aged 19–50) in the USA [17] than for the Puerto Rican women in our study (5.7%). These findings portray a diet in transition, including both the traditional foods as well as contributions from foods that are more commonly part of western-type diets such as that consumed in the USA.

The traditional Puerto Rican diet differs from that of other Hispanic groups in the USA. In our study, as is typical of the Puerto Rican diet, corn and flour tortillas were not large contributors to intake of any nutrient. Tortillas were consumed by only 7% of participants over the 3 days of analysis; most of these servings were from commercial “Mexican-style” fast food restaurants. In Puerto Rico, the dish called “tortilla” is very different; it is an egg-based dish. In another study of diet in Puerto Rico by Palacios et al. [33], a food frequency questionnaire (FFQ) that included traditional Puerto Rican foods was compared to a 6-day food record [34]. For that FFQ, foods were added to a questionnaire developed by the National Cancer Institute, with alterations specific for Puerto Ricans living in the USA [34] and those living in Puerto Rico [33]. Many of the foods that were added to the FFQ for that study included ones that were also frequently reported by the participants in our study: mixed dishes with rice, fritters, fish, avocado, canned meats, plantains, tannier, breadfruit, custard, pudding and cheesecake were among the largest contributors to macronutrients in our study. Other foods such as cranberries, West Indian cherry, Spanish lime, and Mallorca bread which were included in the Palacios et al. [33] FFQ were reported infrequently, if at all, by participants in our study. Participants in the Palacios study were a convenience sample of students, faculty and employees, both males and females, at the Medical Sciences Campus of the University of Puerto Rico [33]. Our study only included women but included a broader sample of the population and a broader age range.

There is less detailed recent information available regarding food sources for macronutrient intake for Hispanics living in the USA [16,25,26,27,28,29,30,34,35,36,37]. There is some evidence that diet quality is poorer among Puerto Ricans in the USA compared to those in Puerto Rico [30], with low intake of fruits and vegetables in Puerto Rico [38]. In our study, the average number of servings of fruit and vegetables was 2.7 per day. Our findings here do not focus on diet quality; rather these data are descriptive of the foods contributing to intake. Among USA Hispanics, intakes differ by ethnic background [29,35], with some evidence of poorer overall diet quality among those of Puerto Rican origin [35]. Among Puerto Ricans in the USA, acculturation may affect dietary intakes [34].

These results provide insight into the diets of women living in Puerto Rico but need to be interpreted in light of the strengths and weaknesses of this study. The use of three separate 24-h recalls, use of the double-pass method of data collection, and provision of serving size pamphlets to participants served to increase the validity of dietary reports. Collection of data by a trained and highly experienced interviewer and the use of the NDSR served to reduce error. Further, the data came from both a list of randomly selected residents and community-based faith groups, providing insight regarding women in the San Juan population. A limitation is that the study was a relatively small one; further studies of this kind including a larger number of individuals would be important for better understanding diet in Puerto Rico. Because the study included only women, these results may not accurately describe the diet of men living in Puerto Rico. In particular, there may be some differences in the order of foods in their contribution to intake for men; it is likely that the foods included in the list are similar. All participants in this study lived in the San Juan metropolitan area. Thus, this study did not capture any regional variation in diet within the island; the results may not generalize to women living in more rural areas. The mean age of study participants was 56.4 years. Diet at this age is of interest because it is the age group typical for age of onset of major chronic diseases including cancer and cardiovascular disease. Nonetheless, these results may not be a representation of the diets of younger women. The group included in the study varied in terms of their characteristics; because of the small sample size it is not possible to use these data to characterize subgroups such as those divided by age or body mass index. Further, a potential source of error in calculation of nutrients was the use of proxy foods to impute nutrient data in the NDSR for a small number of typical Puerto Rican food items for which food composition data were not available. Finally, the groupings of foods in these analyses affected the rankings of their contributions; decisions were made with regard to which foods to group together and which to disaggregate.

## 5. Conclusions

This study of dietary intake of women living in Puerto Rico provides insight into a diet in transition that differs from the USA diet overall and from USA Hispanics. The foods that were important contributors to macronutrient intake in the Puerto Rican diet were a combination of traditional Puerto Rican foods and those characteristic of a Western diet. While many foods important in the Hispanic American diet such as rice and beans were also important in the Puerto Rican diet, other foods commonly consumed by Hispanic Americans such as corn and flour tortillas were not important in the Puerto Rican diet. The diet of these women living in Puerto Rican was a varied one with a large number of foods contributing to intake and variability of energy and macronutrient intake. Identification of foods sources of nutrients for this population with a diet in transition can contribute to the development of instruments to measure dietary intake and to understand its contribution to the etiology of chronic disease among Puerto Rican women. This study also points to the importance of disaggregating dietary intake data for different ethnic and cultural groups categorized as Hispanic or Latino in order to better understand potential diet-related contributions to disparities in chronic diseases.

## Figures and Tables

**Table 1:** Foods contributing to energy (kcal) intake, Puerto Rican women.

Rank	Food Group Name	Percent Contribution to Total Intake	Correlation Coefficient (*r*)	Cumulative	
				Percent Contribution to Total Intake	Variability (*R*^2^)
1	White bread, rolls, crackers	5.5	0.0	5.5	0.0
2	Rice	5.3	−0.1	10.8	0.0
3	Plantain dishes	4.2	0.0	15.0	0.0
4	Rice and vegetable dishes	3.8	0.2	18.8	0.0
5	Reduced fat milk	3.8	0.2	22.7	0.1
6	Chicken, turkey	3.6	0.2	26.3	0.1
7	Cooked cereal	3.5	0.0	29.8	0.1
8	Cakes, cookies, donuts	3.4	0.2	33.2	0.1
9	Cheese	3.1	−0.1	36.3	0.2
10	Beefsteaks, roasts, burgers	3.1	−0.1	39.4	0.2
11	Mixed dishes with chicken	3.0	0.1	42.4	0.2
12	Whole milk, whole milk beverages	2.5	−0.1	44.8	0.2
13	Bean dishes	2.5	0.1	47.3	0.2
14	Pork chops, roasts	2.4	0.1	49.8	0.2
15	Whole grain breads, crackers	2.4	0.0	52.2	0.2
16	Regular soda	2.4	−0.2	54.6	0.2
17	Mixed dishes with beef	2.4	0.1	56.9	0.2
18	Pasta dishes	2.2	0.3	59.2	0.3
19	Mixed dishes with ham or pork	1.9	0.1	61.1	0.3
20	Potatoes, other than French fries	1.9	0.0	63.0	0.3
21	Cold cereal	1.8	0.1	64.8	0.3
22	Sweetened “juice” beverages	1.7	0.1	66.5	0.3
23	French fries	1.5	0.1	68.0	0.3
24	Eggs	1.5	0.0	69.5	0.3
25	Pizza	1.5	0.0	71.0	0.3
26	Tannier, other tuber roots	1.4	0.0	72.4	0.3
27	Margarine	1.4	0.0	73.8	0.3
28	Hotdogs, ham, lunchmeat	1.3	0.2	75.2	0.4
29	Chicken sandwiches	1.3	0.1	76.5	0.4
30	Mixed dishes with seafood	1.2	0.1	77.7	0.4
31	Orange juice	1.2	0.1	78.9	0.4
33	Other fruit juices	1.1	0.0	80.0	0.4
34	Fish, other than fried	0.9	0.0	81.0	0.4
35	White sugar	0.8	−0.1	81.7	0.4
36	Ice cream, frozen desserts	0.8	0.0	82.5	0.4
37	Quesadillas, nachos	0.7	0.0	83.2	0.4
38	Fried chicken	0.7	0.0	84.0	0.4
39	Bananas	0.7	−0.2	84.6	0.4
40	Alcoholic beverages	0.7	−0.1	85.3	0.5
41	Chicken soup	0.7	−0.1	86.0	0.5
42	Apple juice, apple cider	0.6	0.1	86.6	0.5
43	Yogurt	0.6	0.2	87.3	0.5
44	Cornbread, corn tortillas	0.6	−0.1	87.9	0.5
45	Mayonnaise-based dressings, dips	0.5	0.1	88.4	0.5
46	Potato chips, packaged salted snacks	0.5	0.2	88.9	0.6
47	Meal replacement drinks	0.5	0.0	89.4	0.6
48	Lettuce, green salads, greens	0.5	−0.1	89.9	0.6
49	Cheesecake	0.5	0.2	90.4	0.6

**Table 2:** Foods contributing to fat (g) intake, Puerto Rican women.

Rank	Food Group Name	Percent Contribution to Total Intake	Correlation Coefficient (*r*)	Cumulative	
				Percent Contribution to Total Intake	Variability (*R*^2^)
1	Cheese	6.9	0.2	6.9	0.0
2	Beefsteaks, roasts, burgers	4.7	0.3	11.6	0.2
3	Margarine	4.5	0.3	16.0	0.3
4	Cakes, cookies, donuts	4.3	0.2	20.3	0.3
5	Chicken, turkey	4.2	0.2	24.5	0.3
6	Mixed dishes with beef	4.1	0.2	28.6	0.4
7	Pork chops, roasts	4.0	0.2	32.6	0.5
8	Mixed dishes with chicken	3.8	0.2	36.4	0.5
9	Rice and vegetable dishes	3.6	0.1	40.1	0.5
10	Whole milk, whole milk beverages	3.6	0.3	43.6	0.6
11	Reduced fat milk	3.5	0.1	47.2	0.6
12	Plantain dishes	3.3	0.1	50.5	0.6
13	Eggs	3.1	0.1	53.6	0.6
14	White bread, rolls, crackers	2.8	0.1	56.4	0.6
15	Mixed dishes with ham or pork	2.4	0.1	58.8	0.6
16	Cooked cereal	2.4	0.1	61.2	0.7
17	Bean dishes	2.2	0.1	63.4	0.7
18	French fries	2.2	0.2	65.6	0.7
19	Hotdogs, ham, lunchmeat	2.1	0.2	67.7	0.8
20	Mixed dishes with seafood	1.9	0.1	69.6	0.8
21	Pasta dishes	1.8	0.2	71.4	0.8
22	Rice	1.7	0.1	73.2	0.8
23	Pizza	1.6	0.1	74.8	0.8
24	Potatoes, other than French fries	1.6	0.1	76.4	0.8
25	Mayonnaise-based dressings, dips	1.6	0.1	78.0	0.8
26	Chicken sandwiches	1.3	0.1	79.3	0.8
27	Fried chicken	1.1	0.1	80.4	0.9
28	Quesadillas, nachos	1.1	0.1	81.5	0.9
29	Whole grain breads, crackers	1.0	0.0	82.5	0.9
30	Oil-based dressings	1.0	0.1	83.5	0.9
31	Cheesecake	0.9	0.0	84.4	0.9
32	Ice cream, frozen desserts	0.9	0.1	85.2	0.9
33	Lettuce, green salads, green salads	0.8	0.1	86.0	0.9
34	Nuts, seeds	0.8	0.1	86.8	0.9
35	Potato chips, salted packaged snacks	0.8	0.2	87.6	1.0
36	Fish, other than fried	0.8	0.0	88.4	1.0
37	Peanuts, peanut butter	0.7	0.1	89.1	1.0
38	Olive oil	0.6	0.0	89.7	1.0
39	Pies	0.6	0.1	90.4	1.0

**Table 3:** Foods contributing to protein (g) intake, Puerto Rican women.

Rank	Food Group Name	Percent Contribution to Total Intake	Correlation Coefficient (*r*)	Cumulative	
				Percent Contribution to Total Intake	Variability (*R*^2^)
1	Chicken, turkey	12.1	0.2	12.1	0.1
2	Pork chops, roasts	6.6	0.2	18.7	0.2
3	Reduced fat milk	6.5	0.2	25.2	0.3
4	Beefsteaks, roasts, burgers	5.7	0.4	30.9	0.3
5	Cheese	4.5	0.4	35.4	0.3
6	Mixed dishes with beef	3.9	0.1	39.3	0.4
7	Mixed dishes with chicken	3.9	0.2	43.1	0.5
8	Fish, other than fried	3.7	0.2	46.9	0.6
9	White bread, rolls, crackers	3.6	0.0	50.5	0.6
10	Cooked cereal	3.6	0.1	54.0	0.6
11	Bean dishes	3.1	0.2	57.1	0.6
12	Hotdogs, ham, lunchmeat	3.1	0.2	60.2	0.6
13	Whole milk, whole milk beverages	3.0	0.2	63.2	0.7
14	Pasta dishes	2.6	0.2	65.8	0.7
15	Whole grain breads, crackers	2.5	0.1	68.3	0.7
16	Eggs	2.5	0.1	70.8	0.7
17	Mixed dishes with ham or pork	2.4	0.1	73.2	0.8
18	Rice	2.2	0.1	75.4	0.8
19	Chicken sandwiches, wraps	2.2	0.3	77.5	0.8
20	Rice and vegetable dishes	1.7	0.1	79.2	0.8
21	Pizza	1.5	0.1	80.7	0.8
22	Tuna, tuna salad	1.3	0.1	82.0	0.8
23	Mixed dishes with seafood	1.2	0.2	83.3	0.8
24	Cakes, cookies, donuts	1.2	0.0	84.5	0.8
25	Plantain dishes	1.2	0.1	85.7	0.9
26	Cold cereal	1.1	0.1	86.8	0.9
27	Potato, not fried	1.0	0.0	87.8	0.9
28	Fried chicken	0.9	0.1	88.6	0.9
29	Chicken soup	0.8	0.1	89.5	0.9
30	Yogurt	0.8	0.0	90.2	0.9

**Table 4:** Foods contributing to carbohydrate (g) intake, Puerto Rican women.

Rank	Food Group Name	Percent Contribution to Total Intake	Correlation Coefficient (*r*)	Cumulative	
				Percent Contribution to Total Intake	Variability (*R*^2^)
1	White bread, rolls, crackers	7.7	0.1	7.7	0.0
2	Rice	7.5	0.2	15.2	0.0
3	Plantain dishes	6.3	0.2	21.5	0.1
4	Regular soda	4.8	0.2	26.3	0.1
5	Rice and vegetable dishes	4.5	0.2	30.8	0.2
6	Cooked cereal	4.2	0.3	35.0	0.3
7	Cakes, cookies, donuts	3.6	0.2	38.7	0.3
8	Sweetened “juice” beverages	3.4	0.2	42.0	0.4
9	Whole grain breads, crackers	3.2	0.2	45.3	0.4
10	Reduced fat milk	3.0	0.1	48.3	0.4
11	Cold cereal	3.0	0.3	51.3	0.5
12	Tannier, other tuber roots	2.7	0.1	54.0	0.5
13	Bean dishes	2.5	0.1	56.5	0.6
14	Potatoes, other than French fries	2.4	0.2	58.8	0.6
15	Pasta dishes	2.4	0.2	61.2	0.6
16	Orange juice	2.2	0.1	63.4	0.6
17	Mixed dishes with chicken	2.1	0.2	65.5	0.6
18	Other fruit juices	2.1	0.1	67.6	0.6
19	White sugar	1.7	0.2	69.3	0.7
20	Whole milk, whole milk beverages	1.3	0.0	70.9	0.7
21	French fries	1.5	0.2	72.4	0.7
22	Mixed dishes with ham or pork	1.4	0.2	73.8	0.7
23	Pizza	1.4	0.1	75.2	0.7
24	Bananas	1.3	0.2	76.5	0.8
25	Apple juice, apple cider	1.2	0.1	77.7	0.8
26	Beefsteaks, roasts, burgers	1.0	0.1	78.8	0.8
27	Chicken sandwiches	1.0	0.0	79.8	0.8
28	Ice cream, frozen desserts	1.0	0.1	80.8	0.8
29	Apples, apple sauce	0.9	0.2	81.7	0.8
30	Yogurt	0.8	0.1	82.5	0.8
31	Mixed dishes with seafood	0.8	0.1	83.3	0.8
32	Mixed dishes with beef	0.7	0.1	84.0	0.9
33	Cornbread, corn tortillas	0.7	0.1	84.8	0.9
34	Mango	0.7	0.1	85.5	0.9
35	Chicken soup	0.6	0.0	86.1	0.9
36	Meal replacement drinks	0.6	0.1	86.7	0.9
37	Vegetable soup	0.6	0.1	87.3	0.9
38	Candy	0.6	0.1	87.9	0.9
39	Quesadillas, nachos	0.5	0.0	88.4	0.9
40	Honey, jams, jellies, syrup	0.5	0.0	88.9	0.9
41	Grapefruit juice	0.5	0.1	89.4	0.9
42	Potato chips, packaged salted snacks	0.5	0.1	89.9	0.9
43	Pies	0.5	0.0	90.3	0.9

**Table 5:** Foods contributing to dietary fiber (g) intake, Puerto Rican women.

Rank	Food Group Name	Percent Contribution to Total Intake	Correlation Coefficient (*r*)	Cumulative	
				Percent Contribution to Total Intake	Variability (*R*^2^)
1	Bean dishes	11.0	0.3	11.0	0.1
2	Plantain dishes	7.8	0.3	18.9	0.2
3	Tannier, other tuber roots	6.5	0.4	25.4	0.4
4	Whole grain breads, crackers	6.5	0.4	31.8	0.5
5	White bread, rolls, crackers	5.5	0.1	37.4	0.5
6	Cooked cereal	4.1	0.3	42.2	0.6
7	Potatoes, other than French fries	3.5	0.1	45.7	0.6
8	Pasta dishes	2.8	0.2	48.5	0.7
9	Lettuce, green salads, greens	2.6	0.1	51.2	0.7
10	French fries	2.5	0.1	53.6	0.7
11	Bananas	2.4	0.1	56.1	0.7
12	Cold cereal	2.0	0.2	58.0	0.7
13	Apples, applesauce	1.9	0.2	59.9	0.8
14	Cakes, cookies, donuts	1.8	0.1	61.7	0.8
15	Mixed dishes with chicken	1.7	0.0	63.4	0.8
16	Rice	1.7	0.1	65.1	0.8
17	Rice and vegetable dishes	1.6	0.1	66.7	0.8
18	Mixed dishes with beef	1.4	0.0	68.2	0.8
19	Pears	1.4	0.1	69.6	0.8
20	Chicken sandwiches, wraps	1.4	0.0	71.0	0.8
21	Pizza	1.3	0.1	72.3	0.8
22	Peas	1.2	0.0	73.6	0.8
23	Mango	1.2	0.2	74.7	0.9
24	Beefsteaks, roasts, burgers	1.2	0.1	75.9	0.9
25	Mixed dishes with seafood	1.2	0.1	77.1	0.9
26	Sweet potatoes, yams	1.1	0.0	78.2	0.9
27	Orange juice	1.1	0.0	79.3	0.9
28	Oranges	0.9	0.1	80.2	0.9
29	Mixed dishes with ham or pork	0.8	0.1	81.0	0.9
30	Other fruit juices	0.8	0.0	81.8	0.9
31	Nuts, seeds	0.8	0.0	82.5	0.9
32	Cornbread, corn tortillas	0.8	0.1	83.3	0.9
33	Mixed fruit, other fruits not listed	0.8	0.1	84.1	0.9
34	Sweetened “juice” beverages	0.7	0.0	84.8	0.9
35	Vegetable soup	0.7	0.0	85.5	0.9
36	Chicken soup	0.6	0.0	86.1	0.9
37	Quesadillas, nachos	0.6	0.1	86.7	0.9
38	Cabbage, coleslaw	0.6	0.0	87.3	0.9
39	Yogurt	0.6	0.1	87.8	0.9
40	Other vegetables	0.6	0.1	88.4	0.9
41	Tomatoes	0.6	0.0	89.0	0.9
42	Potato chips, packaged salted snacks	0.5	0.1	89.5	1.0
43	Meal replacement shakes	0.5	0.0	90.0	1.0

**Table 6:** Prevalence of Consumption of Foods, Puerto Rican Women.

Rank	Food Name	Prevalence (%)
1	White breads, rolls, crackers	82
2	Cheese	82
3	Coffee, tea	77
4	Rice	72
5	Bean dishes	68
6	Ice cream, frozen desserts	67
7	Chicken, turkey	63
8	Candy	63
9	Cakes, cookies, donuts	60
10	Hot dogs, ham, lunchmeat	58
11	Reduced fat milk	57
12	Margarine	56
13	Lettuce, green salads, greens	55
14	Regular soda	54
15	Whole milk, whole milk beverages	50
16	Artificial sweeteners	49
17	Cooked cereal	48
18	Whole grain breads, crackers	47
19	Eggs	47
20	Plantain dishes	45
21	Sweetened “juice” beverages	44
22	Cold cereal	44
23	Rice and vegetable dishes	43
24	Potatoes, other than French fries	41
25	White sugar	39
26	Assorted condiments	35
27	Mixed dishes with chicken	35
28	Pork chops, roasts	35
29	Orange juice	34
30	Other fruit juices	34
31	Tomatoes	32
32	Pies	30
33	Chicken soup	27
34	Fish, other than fried	27
35	Mixed dishes with beef	27
36	Beefsteaks, roasts, burgers	27
37	Pasta dishes	26
38	Tannier, other tuber roots	25
39	Mixed dishes with seafood	24
40	Mayonnaise-based dressings, dips	24
41	Diet soda	23
42	Bananas	23
43	Mixed fruit, other fruits not listed	22
44	French fries	22
45	Yogurt	20
46	Mixed vegetables, other vegetables not listed	19
47	Mixed dishes with ham or pork	18
48	Onions, garlic	18
49	Vegetable soup	16
50	Chicken sandwiches, wraps	16
51	Apples, applesauce	16
52	Oil-based dressings	15
53	Pizza	15
54	Corn	14
55	Carrots	14
56	Apple juice, apple cider	13
57	Tuna, tuna salad	13
58	Alcoholic beverages	13
59	Peas	13
60	Coleslaw, cabbage	13
61	Honey, jams, jellies, syrup	12
62	Nuts, seeds	11
63	Avocado, guacamole	11
64	Potato chips, packaged salted snacks	10
65	Olive oil	10
66	Sweet potatoes, yams	10
67	Mango	10
68	Green beans	9
69	Skim milk	9
70	Peanuts, peanut butter	8
71	Oranges	8
72	Broccoli	8
73	Grapefruit juice	7
74	Carrot juice	7
75	Fried chicken	7
76	Hot chocolate	7
77	Flour tortillas	7
78	Cucumber	7
79	Yucca	6
80	Squash	6
81	Pears	6
82	Papaya	6
83	Grapes	6
84	Meal replacement drinks	5
85	Cheesecake	5
86	Bacon	5
87	Plums	5
88	Pineapple	5
89	Fried fish	4
90	Shellfish	4
91	Popcorn	4
92	Berries	4
93	Soymilk	3
94	Sausages	3
95	Peaches, nectarines	3
96	Gelatin	2
97	Meat substitute	1
98	Butter	1
